# Editorial: Machine Learning for Non/Less-Invasive Methods in Health Informatics

**DOI:** 10.3389/fdgth.2021.763109

**Published:** 2021-10-06

**Authors:** Kun Qian, Liang Zhang, Kezhi Li, Juan Liu

**Affiliations:** ^1^School of Medical Technology, Beijing Institute of Technology, Beijing, China; ^2^School of Computer Science and Technology, Xidian University, Xi'an, China; ^3^Xi'an Key Laboratory of Intelligent Software Engineering, Xidian University, Xi'an, China; ^4^Institute of Health Informatics (IHI), University College London (UCL), London, United Kingdom; ^5^Department of Plastic Surgery, Central Hospital of Wuhan, Tongji Medical College, Huazhong University of Science and Technology, Wuhan, China

**Keywords:** digital health, medicine 4.0, intelligent medicine, machine learning, deep learning, artificial intelligence, non/less-invasive methods

## 1. Introduction

At the time of writing this editorial, COVID-19, as an unprecedented pandemic, has caused more than 4.4 million people left us foreverdeaths worldwide (with more than 210 million confirmed cases) in the world^1^. As researchers, this fact urges us to think about how to leverage the power of advanced technologies in improving the life quality of human beings and fighting against the ongoing and/or future pandemic. In particular, the core technology of artificial intelligence (AI), i. e., machine learning (ML) (1), has been playing an increasingly important role in leading the frontiers of Medicine improving the field of medicine 4.0.

In recent years, non/less-invasive methods are have been fast developing in clinical practice, which can considerably reduce the pains and burdens to patients physiologically and psychological pain of patiently. On the one hand, benefited from the breakthroughs in big data, the internet of things (IoT), 5G, cloud computing, high performance computing (HPC), and wearable sensors, and other AI-enabled methods have been successfully applied to tremendous scenarios such as diagnose, treatment, and management of diseases, assisted living, and rehabilitation training. On the other hand, there are existing challenges and technical and ethical issues that need to be addressed. To this end, we organized a research topic entitled “*Machine Learning for Non/Less-Invasive Methods in Health Informatics*” to build an open forum for scientists, engineers, and clinicians to exchange their studies, insights, and perspectives via a multidisciplinary point of view. The collection work lasted for 1 year (from February 2020 to February 2021), and finally it leadsled to 16 articles accepted and published after the peer-reviewed process. There are 127 authors involved in this research topic which has attracted more than 22 000 views (to as of September 2021).

In the following parts of this editorial, we will make a brief description of the published research articles within this research topic. After that we give our perspectives toward future work.

## 2. Data Modalities

**Figure 1:**
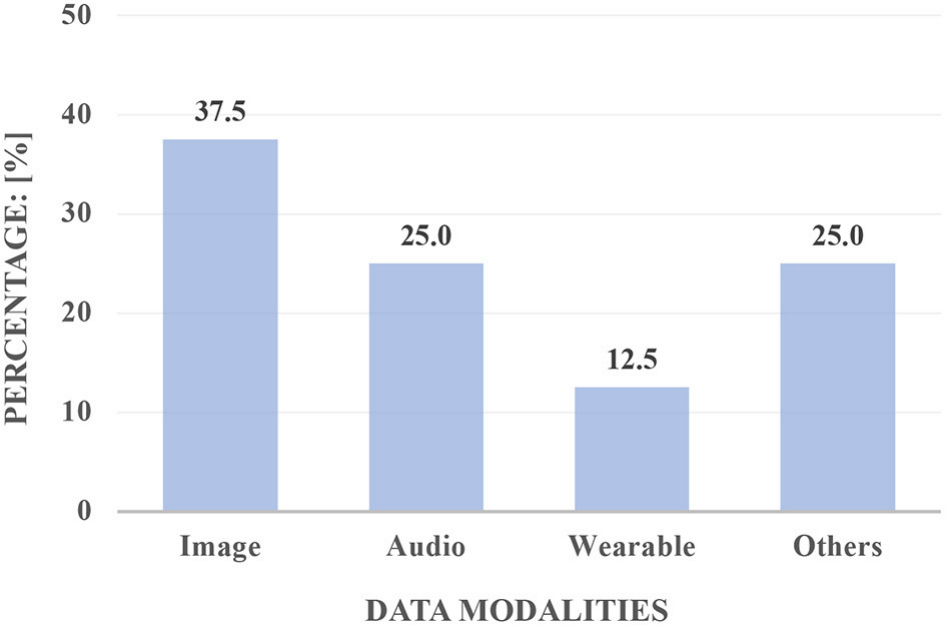
The proportion (in [%]) of the articles in our research topic by viewing the data modalities.

Figure 1 shows the proportion of articles that used one kind of data modality in our collected contributions. We can find that medical imaging dominated in the application, which is related to computer vision (CV).

### 2.1. Image

Aging population has Aging populations have become an inevitable challenge for both developing and developed countries, which and is continuously attracting efforts from the community of AI and IoT (2). The early diagnosis of brain diseases, e. g., Alzheimer's disease (AD) (3), can be very much important essential for benefiting guaranteeing a safe, easy, and independent life for the elderly, particularly for those who are living alone. Song et al. proposed a multimodal image fusion method that combines the representations learnt from the magnetic resonance imaging (MRI) (4) and the positron emission tomography (PET) (5). In their method, both the contour and the metabolic characteristics of the subject's brain tissue are retained.

Diagnosis of cancer via imaging has always been regarded as a crucial computer-aided form of medical technology. Li et al. built a dataset of pulmonary lesions with multiple-level attributes and fine contours. Wang et al. contributed two articles in their recent studies on tumor segmentation: One used octave convolutions to learn multiple-spatial-frequency features from the computed tomography (CT) (6) images for liver tumor segmentation. The other one proposed a framework of multi-modalities interactive feature learning for brain tumor segmentation.

A hierarchical deep learning (DL) (7) network was proposed by Hong et al. in their work for diagnosing multiple visual impairment diseases. A family of multi-task and multi-label learning classifiers was employed to represent different levels of eye diseases. Forte et al. proposed a DL method for identification of acute illness and facial cues of illness. Interestingly, their experiments demonstrated that the synthetically generated data can be used to develop algorithms for health conditions.

### 2.2. Audio

Compared to its counterpart, CV, computer audition (CA) has been underestimated for a long time in the field of digital health. Nevertheless, audio as a novel digital phenotype, is attracting more attention in recent decades than ever before (8). Specifically, the analysis of cough sound has been found to be efficient in for an early-diagnosis of COVID-19 (9). Hou et al. proposed a novel feature set based on non-linear acoustic characteristics extracted from the snore sound. Their method can be used for estimating the severity levels of the obstructive sleep apnoeaapnea (OSA) (10). Li and Tian proposed an unsupervised learning method based on variational auto-encoders (VAEs) for detection of abnormal heart sounds. Yang et al. shared their clinical opinions of CA- based methods for bowel sound analysis and its potential in diagnosis of intestinal obstruction. Besides the aforementioned physiological diseases, audio can also be applied to the diagnosis of psychiatric diseases. For instance, Zhang et al. proposed a speech emotion recognition framework based on pre-trained attentive convolutional neural network, which may be adopted for developing a speech-driven method for detection of depression.

### 2.3. Wearable

Li et al. studied the ML-based models for estimating the associations between the body accelerations and the large-scale objective sleep data. Their study contributed to an objective evaluation of sleep quality by considering the seasonal changes in meteorological factors (e. g., ambient temperature, humidity, and sunlight). Ishaque et al. showed us a review on analyzing the heart rate variability (HRV) data and its associations in to morbidity, pain, drowsiness, stress, and exercise via signal processing (SP) and ML methods.

### 2.4. Others

Guo et al. used ML and DL models to predict the proximity to catastrophic decompensation from the synthetic electronic health record (EHR) data. This method can improve the timing of high-risk heart failure (HF) (11) surgical intervention. Elgendi et al. showed that unsupervised learning models can be used to reveal the novel correlates of chronic pelvic pain (CPP) (12) in women. Zhu et al. implemented ML models for predictingto predict the central lymph node metastasis in T1-T2, non-invasive, and clinically node negative papillary thyroid carcinoma (13). Sang et al. introduced a model using blood markers and logistic regression for diagnosis of fibrosis in southeast Asian patients suffering from the non-alcoholic fatty liver disease (NAFLD) (14).

## 3. Perspectives

It is encouraging to see the state-of-the-art ML models are being successfully applied to the field of non/less-invasive methods in health informatics. Nevertheless, we understand that there still exist several challenges: First, the data scarcity is restraining the reproducibility and sustainability of the relevant studies. Taking bowel sound analysis work as an example, the publicly accessible database is extremely limited. There is an urgent demand for future collaborations between experts in AI and medicine to build open access databases. Second, breaking the walls between disciplines can never be an easy work. When reading the articles written by authors from different backgrounds, we may find there are limitations and drawbacks caused by knowledge frontiers. For instance, computer scientists can be more professional than clinicians in conducting a good ML/DL experiment whereas the latter may be clearer than the former about the motivation and the significance of the proposed research. Basic knowledge and skills training is a prerequisite for future training of experts in digital health. Third, multi-modal learning has already shown its superior performance to models trained by mono-modal. In future work, one should take image, audio, wearable, and other possible modalities into account when studying the complex associations between diseases and subjects' health data. Last but not least, ethical issues were not fully discussed in this research topic collection. We cannot ignore this important factor when working toward a human-centredcentered medical AI. Experts from social and humanity sciences are very welcome to be on board with usto collaborate with us.

## Author Contributions

KQ drafted the first version of this editorial and the other co-authors contributed to the proofreading work. All the co-authors contributed to this work.

## Funding

This work was supported in part by the BIT Teli Young Fellow Program from the Beijing Institute of Technology, China, in part by the National Natural Science Foundation of China under grant nos. 62073258 and 62072352, and in part by the National Key R&D Program of China under grant no. 2020YFF0304900, UK Rosetrees Trust UCL-IHE-2020-102 and GOSH NHS Foundation Trust DRE.

## Conflict of Interest

The authors declare that the research was conducted in the absence of any commercial or financial relationships that could be construed as a potential conflict of interest.

## Publisher's Note

All claims expressed in this article are solely those of the authors and do not necessarily represent those of their affiliated organizations, or those of the publisher, the editors and the reviewers. Any product that may be evaluated in this article, or claim that may be made by its manufacturer, is not guaranteed or endorsed by the publisher.

## Footnotes

^1^https://coronavirus.jhu.edu/map.html.

## References

[1] Bishop CM. Pattern Recognition and Machine Learning. New York, NY: Springer (2006).

[2] Qian K Zhang Z Yamamoto Y Schuller BW. Artificial intelligence internet of things for the elderly: from assisted living to health-care monitoring. IEEE Signal Proc Mag. (2021) 38:78–88. 10.1109/MSP.2021.3057298

[3] Khachaturian ZS. Diagnosis of Alzheimer's disease. Arch Neurol (1985) 42:1097–105. 10.1001/archneur.1985.04060100083029 2864910

[4] Geva T. Magnetic resonance imaging: historical perspective. J Cardiovasc Magn Reson. (2006) 8:573–80. 10.1080/10976640600755302 16869310

[5] Ollinger JM Fessler JA. Positron-emission tomography. IEEE Signal Proc Mag. (1997) 14:43–55.

[6] Fleischmann D Boas FE. Computed tomography–old ideas and new technology. Eur Radiol. (2011) 21:510–7. 10.1007/s00330-011-2056-z 21249371

[7] LeCun Y Bengio Y Hinton G. Deep learning. Nature. (2015) 521:436–44. 10.1038/nature14539 26017442

[8] Qian K Li X Li H Li S Li W Ning Z. Computer audition for healthcare: opportunities and challenges. Front Digit Health. (2020) 2:5. 10.3389/fdgth.2020.00005 34713018 PMC8521830

[9] Qian K Schuller BW Yamamoto Y. Recent advances in computer audition for diagnosing COVID-19: an overview. In: 2021 IEEE 3rd Global Conference on Life Sciences and Technologies (LifeTech) (2021) Nara. p. 185–6.

[10] Strollo Jr PJ Rogers RM. Obstructive sleep apnea. N Engl J Med. (1996) 334:99–104. 8531966 10.1056/NEJM199601113340207

[11] Kemp CD Conte JV. The pathophysiology of heart failure. Cardiovasc Pathol. (2012) 21:365–71. 10.1016/j.carpath.2011.11.007 22227365

[12] Howard FM. Chronic pelvic pain. Obstet Gynecol. (2003) 101:594–611. 10.1016/s0029-7844(02)02723-0 12636968

[13] Sherma SI. Thyroid carcinoma. Lancet. (2003) 361:501–11. 10.1016/s0140-6736(03)12488-9 12583960

[14] Bellentani S Scaglioni F Marino M Bedogni G. Epidemiology of non-alcoholic fatty liver disease. Dig Dis. (2010) 28:155–61. 10.1159/000282080 20460905

